# Factors Contributing to the Link between Physical Well-Being and Chronic Pain in Young People from Galicia, Northwest Spain

**DOI:** 10.3390/jcm12134228

**Published:** 2023-06-23

**Authors:** Roya Karimi, Jesús Prego-Domínguez, Bahi Takkouche

**Affiliations:** 1 Department of Preventive Medicine, University of Santiago de Compostela, 15782 Santiago de Compostela, Spain; royakarimi.karimi@usc.es (R.K.); susofis@gmail.com (J.P.-D.); 2 Centro de Investigación Biomédica en Red de Epidemiología y Salud Pública (CIBER-ESP), 28029 Madrid, Spain

**Keywords:** chronic pain, confounding, interaction analysis, mediation analysis, physical well-being

## Abstract

**Introduction:** The relation between physical well-being and chronic pain is complex and involves several subjective and objective covariates. We aimed to assess the role of mediator, confounder, or interactor played by covariates, including sleep quality, physical activity, perceived stress, smoking, and alcohol drinking in the relation between physical well-being and chronic pain. **Method:** We used Poisson regression to obtain incidence rate ratios (IRR) of the association between physical well-being and chronic pain in a cohort study carried out among university students. We applied General Structural Equation Modeling (GSEM) to assess mediation and stratum-specific analyses to distinguish confounding from interaction. We computed Relative Excess Risks due to Interaction (RERI), Attributable Proportion (AP), and the Synergy index (S) to measure additive interaction. **Results:** High physical well-being is related to a large decrease in the risk of chronic pain (IRR^Total Effect^ = 0.58; 95% CI: 0.50–0.81). Perceived stress mediates 12.5% of the total effect of physical well-being on chronic pain. The stratum-specific IRRs of current smokers and non-current smokers were different from each other and were larger than the crude IRR (IRR = 1.49; 95% CI: 1.24–1.80), which indicates that smoking could be both confounder and interactor. Interaction analyses showed that physical activity could act as a potential interactor (RERI = 0.25; 95% CI: 0.13, 0.60). **Conclusions:** Perceived stress is an important mediator of the relation between physical well-being and chronic pain, while smoking is both a confounder and an interactor. Our findings may prove useful in distinguishing high-risk groups from low-risk groups, in the interventions aimed at reducing chronic pain.

## 1. Introduction

Chronic pain is a psychophysiological perception in which somatosensory inputs convert into the physiological, cognitive, affective, and functional responses identified as chronic pain [1]. Pain is usually considered a symptom or indicator of underlying diseases. However, due to its notable influence on quality of life and its economic burden, chronic pain was defined in the last decades as an illness “in its own right” [2]. Chronic pain, resulting from musculoskeletal conditions, is one of the most common chronic health issues affecting the global population [3,4]. Globally, one in every nine young adults suffers from it [5] and out of 139 million disability-adjusted life years (DALY) accounted for by musculoskeletal disorders, 65 million are due to low back pain [6].

It was estimated that approximately 36% of adolescents in Spain experience chronic pain [7]. Furthermore, the Spanish National Health Survey showed that chronic back pain alone had a prevalence of almost 24% in the general Spanish population and that this prevalence is higher (30%) in Galicia, the region in which the present study was carried out [8]. 

Living in a humid climate was suggested as a potential risk factor for chronic pain occurrence [9]. It happens that Galicia has the highest relative humidity of all Spanish regions [10]. Recently, a study in the United Kingdom revealed that individuals experiencing chronic pain tend to report higher pain levels on days with increased humidity [11]. Furthermore, Galicia was found to be the region with the lowest well-being values in Spain [12]. The average frequency of self-perceived poor health in Spain was reported to be 33.6%, while the highest frequency was 45.7%in Galicia [13]. 

Physical well-being is a multidimensional concept that encompasses physical, mental, social, cultural, and cognitive dimensions [14]. Physical well-being and chronic pain represent interrelated multidimensional concepts with highly subjective components [15,16]. The relation between physical well-being with chronic pain is not straightforward. For instance, it is remarkable that patients with impairments or diseases do not necessarily report poor physical well-being [17]. 

The relation of well-being with chronic pain is either *mediated* by factors in the causal pathway (indirect effect) or *unmediated* (direct effect). Other factors may also play the role of *confounders* of the relation between well-being and pain. To be a confounder, a variable should associate with both exposure and outcome and distort the association between them [18]. Modern epidemiology shows that, to be a mediator, a variable should be in the pathways between exposure and outcome and account for their association, either partially or entirely [19]. Furthermore, *interaction* occurs when two factors collaborate to produce an outcome, i.e., when the presence of either of them influences the effect of the other. Improving knowledge about the joint effects of covariates with physical well-being in causing chronic pain aids in identifying high-risk subgroups.

To assess the role of mediator, confounder, or interactor played by the variables of sleep quality, physical activity, perceived stress, smoking, and alcohol drinking in the relationship between physical well-being and chronic pain, we analyzed the data of a prospective cohort study carried out in Spain. Assessing the causal role of each covariate provides insight into the high prevalence and incidence of chronic pain in Galicia.

## 2. Method

### 2.1. Study Sample and Procedure

The Pain Study Online is a web-based follow-up study aiming to identify risk factors for chronic pain in a young population. 

In 2019, 2000 students of a Galician university were invited to complete a baseline questionnaire on demographic, behavioral, medical, and nutritional aspects, as well as pain measurements. The sample size of the study was estimated using the instructions of Wang and Ji [20] with power = 80%, type 1 error rate = 0.05, the expected probability of chronic pain in the unexposed group = 0.30, the ratio of unexposed to exposed = 3, and the Incidence Rate Ratio (IRR) to be detected = 1.5. Assuming a dropout proportion of 0.2, the total necessary sample size was 948. 

The exposure and covariates were collected at baseline, and pain episodes were detected using a questionnaire sent every four months during one-year of follow-up. 

The University of Santiago de Compostela institutional review board approved this study (reference BT-RDC-2017-01). Informed consent was obtained from each participant before starting the study.

### 2.2. Outcome Definition

The outcome was defined, using the International Association for the Study of Pain (IASP) definition [21], as pain at any part of the body that lasts more than three months, and was assessed by the question: “In the past four months, did you have pain that lasted for more than three months?” If the answer was “yes”, participants had to specify which part of their body was affected by chronic pain and then answer the Short-Form McGill Pain Questionnaire 2 (SF-MPQ-2), validated in Spain, in order to describe the characteristics of the pain.

### 2.3. Exposure Definition

Information on physical well-being, the exposure variable of this study, was obtained from the short-form 12-item health survey (SF-12) validated in the Spanish population [22]. This survey evaluates two components, mental and physical, of Health-Related Quality of Life (HRQoL) [22,23]. It generates a score between 0 and 100, calculated using algorithms and recommended coefficients, with higher scores indicating better well-being [24]. For our purpose, we subsequently transformed the calculated scores into a binary variable: lower/higher than the median score.

### 2.4. Covariate Definition

Demographic characteristics (age and sex) and lifestyle habits were collected at baseline using a self-administered and validated questionnaire. 

Smoking status was divided into three categories: Current smokers (subjects who smoked more than one cigarette per week in the past six months), former smokers (subjects who previously smoked at least one cigarette per week but had quit more than six months ago), and non-smokers (subjects who had not smoked any cigarettes). In our analysis, for practical reasons, we combined non-smokers and former smokers into a binary variable: “non-current smokers” and “current smokers”.

Alcohol consumption information was collected from a food frequency questionnaire [25] and was categorized, based on the Centers for Disease Control and Prevention guidelines, into abstainers/low drinkers (subjects who either reported no alcohol consumption or drank less than once a day for women and twice or less than twice a day for men) and moderate drinkers (subjects who drank alcohol once a day for women and twice a day for men). Subjects with an intake higher than moderate were exceptional in our population (10 subjects only) and, therefore, were excluded from analysis.

A score of physical activity was obtained using the short form of the International Physical Activity Questionnaires (IPAQ-SF) to define two groups, low and high activity, based on pre-established algorithms [26]. 

Sleep quality was assessed using the Pittsburgh Sleep Quality Index (PSQI). The calculated scores, ranging from 0 to 21, with lower scores indicating higher sleep quality, were divided into two categories using a cut-off value of 6, the median score of the sample [27]. 

Perceived stress was considered a dichotomous variable using a cut-off value of 8, the median score obtained from the Perceived Stress Scale questionnaire (PSS-4) [28]. Higher scores indicate more perceived stress.

All questionnaires were previously validated in the Spanish population, and the baseline questionnaire was pilot-tested in the study population before the study initiation. Those individuals who participated in the pilot test did not form part of the cohort study.

### 2.5. Measures of Association

Person-time was calculated as the time elapsed from the date of filling out the baseline questionnaire until the onset of a chronic pain episode, loss to follow-up, or end of the study, whichever appeared first. Cases of chronic pain were allocated half of the period between the last follow-up and the onset of the episode, assuming constant incidence during that period. 

To examine the relation between physical well-being and chronic pain occurrence, incidence rate ratios (IRR) and their 95% confidence intervals (95% CI) were estimated by Poisson regression. 

**Figure 1:**
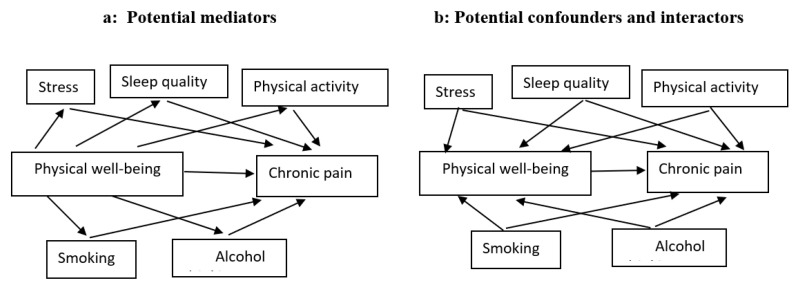
A hypothesized causal diagram of the model with covariates of the association between physical well-being and chronic pain.

We used directed acyclic graphs (DAGs) to represent our hypothesis and assumptions regarding the causal relationship between physical well-being and chronic pain (Figure 1). Two types of covariates considered in our models are confounders (variables that share causes of the exposure and outcome) and mediators (variables that transmit some of the effect of interest through an indirect pathway). Colliders (variables that are caused by the outcome and the exposure) were not considered further, due to the longitudinal design of our study that forces exposure and covariates to precede outcome. We also represented interaction covariates (also known as interactors or moderators), which are variables that interact with exposure to affect the outcome. Since DAGs are nonparametric representations, interactors/moderators are depicted in the same way as confounders. However, we distinguished the interactors from confounders when we translated the DAG into a statistical model, using stratum-specific analysis (0).

### 2.6. Mediation Analysis

We used General Structural Equation Modeling (GSEM) to test whether the potential mediators (physical activity, perceived stress, sleep quality, smoking status, and alcohol consumption) may represent indirect causal paths between physical well-being (main exposure) and chronic pain (Figure 1). We first estimated the model parameters and causal effects, including Natural Direct Effect (NDE), Natural Indirect Effect (NIE), and Total Effect (TE). The indirect effects were generated by multiplying the estimated regression coefficient of physical well-being on each mediator by the regression coefficient of each mediator on chronic pain [29]. The direct effects were generated by the regressions of the association between physical well-being and pain perception. Finally, the total effects comprised a sum of direct and indirect effects captured in GSEM analysis [30]. We calculated the proportion mediated as (IRR_NDE_ × [IRR_NIE_ − 1])/(IRR_NDE_ × IRR_NIE_ − 1).

### 2.7. Stratum-Specific Analysis

In order to distinguish confounding from interaction (Figure 1b), we separately measured the relation between physical well-being and pain in the different strata of each covariate (perceived stress, smoking, alcohol consumption, and sleep quality). If the crude overall estimate and the stratum-specific estimates were similar, the covariate was considered neither a confounder nor an interactor. If the stratum-specific measures of association were *similar* to each other, but differed from the overall crude estimate by 10% or more [31], we considered this covariate as a confounder. If the stratum-specific estimates *differed* from each other, we considered this covariate as an interactor. There is both confounding and interaction if (1) the stratum-specific estimates vary from each other and *both* have higher or lower values than the crude estimate, or (2) the stratum-specific estimates differ from each other, and the crude estimate is *between* the two stratum-specific estimates. In the latter case, we adjusted the Poisson regression estimate for the covariate to find out whether the adjusted estimate differs from the crude estimate by more than 10%. Those covariates that changed crude IRR estimates by more than 10% were considered both confounders and interactors. If the crude estimate did not differ by more than 10%, we considered the covariate to be only an interactor.

### 2.8. Interaction Analysis

After performing stratum-specific analysis and deciding on the confounding and interactional nature of the covariates, we performed an additive interaction analysis for the variables that were not potentially eligible as confounders of the association between physical well-being and chronic pain.

Additive interaction analyses between physical well-being and each covariate were performed to measure the joint effects of the combination of two potential risk factors [31]. For each physical well-being–covariate relation, we computed the adjusted Relative Excess Risk due to Interaction (RERI), also named Interaction Contrast Ratio (ICR), the Attributable Proportion (AP), and the Synergy index (S), along with their 95% CIs [32,33]. 

RERI is interpreted as the additional risk due to interaction added to the expected total risk by separately summing each exposure’s risks. The AP is interpreted as the proportion of the outcome due to the interaction of both exposures. Accordingly, an RERI and AP > 0 imply a deviation from additivity and provide evidence that the exposure and the covariate may have a joint effect in causing the outcome [34]. The low-risk groups were considered as the reference category. However, since moderate alcohol consumption is known to exert analgesic effects [35], in this study, we considered moderate alcohol consumption as the reference category. The joint effect analyses were adjusted for age and sex. We used Poisson regression to estimate IRRs of chronic pain and their 95% CIs for interaction analysis. 

All the analyses were conducted on multiply imputed data. In this study, to generate more precise estimates, we imputed the missing data first and then performed the analysis and bootstrapping, as explained hereafter [36]. Ten imputed data sets were generated via Multiple Imputation by Chained Equations (MICE), and a fitted conditional imputation model was obtained for each variable used for imputation. The analysis was replicated for each multiply imputed data set, and the estimates were pooled from the 10 imputed data sets [37]. We used bootstrap resampling to calculate the mean point estimate for the parameters by repeating mediation analysis across 1000 bootstrapped data sets [38]. Bias-corrected and accelerated 95% CIs were calculated to assess skewness in the confidence interval obtained from mediation analysis [39].

All analyses were performed with STATA/MP software version 15.1 (Stata Corp LLC, TX, USA). 

## 3. Results

### 3.1. Characteristics of the Study Population

**Figure 2:**
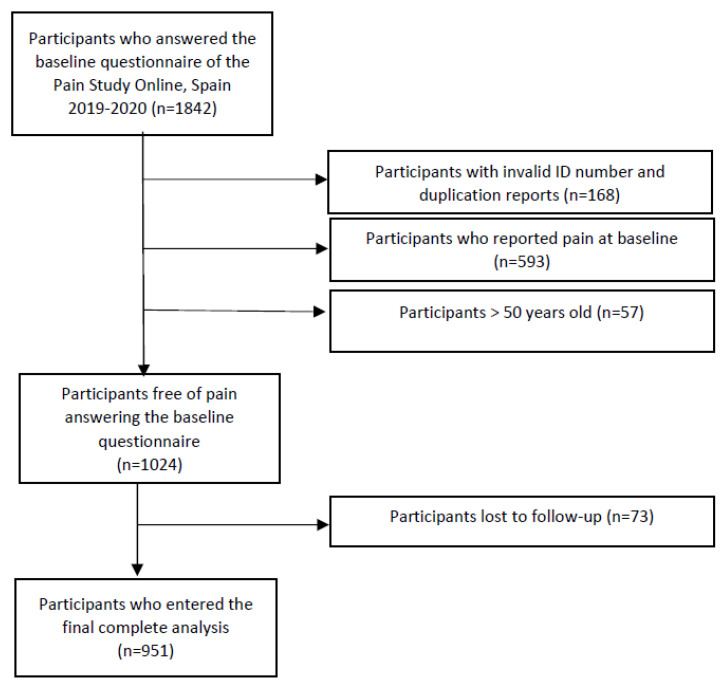
Flow diagram of Pain Study Online, Spain, 2019–2020.

In total, 1842 students completed the baseline questionnaire. After excluding participants aged >50, those who did not provide any ID or were duplicated, and subjects who reported pain at baseline, a total of 1024 students were free of pain at the beginning of the study and were then enrolled in the follow-up (Figure 2). However, 73 were lost to follow-up, so 951 were finally included in the analysis. During the follow-up, we identified 584 new pain cases, with a total of 160.4 years at risk, which yielded an overall incidence rate of 3.6 year^−1^.

Table 1 presents the study population’s descriptive results by physical well-being status for all variables. The mean score of physical well-being was 55.63 (SD: 5.44), and the population was evenly distributed across sex and age groups, with a mean age of 25.29 ± 5.7 and 24.31 ± 6.3 years for men and women, respectively. In addition, the baseline physical well-being scores were similar in women and men (mean = 55.7 and 55.1, respectively). 

### 3.2. Mediation Analysis

Mediation analysis was separately conducted for all potential mediators. Figure 1a displays our hypothesized relations, and the estimated total causal effect, as well as the direct and indirect effects of physical well-being on chronic pain, is shown in Table 2.

GSEM analysis revealed that, compared to low physical well-being, high physical well-being is related to a large decrease in the risk of chronic pain (IRR^Total Effect^ = 0.58; 95% CI: 0.50–0.81) and that the indirect effect of the perceived stress of that relation was IRR^Natural Indirect Effect^ = 0.92; 95% CI: 0.89–1.00. Perceived stress mediates 12.5% of the total effect of physical well-being on chronic pain (Table 2). Other potential mediators, including physical activity, smoking, drinking, and sleep quality, showed a 6.4%, 4.8%, and 3.4% mediation proportion of the association between physical well-being and chronic pain.

### 3.3. Stratum-Specific Analysis

Table 3 shows the results of stratum-specific analysis. The crude estimate value (IRR = 1.49; 95% CI: 1.24–1.80) was between the low physical activity value (IRR = 1.76; 95% CI: 1.28–2.36) and the high physical activity value (IRR = 1.20; 95% CI: 1.06–1.45), and the adjusted estimate did not change the crude estimate by more than 10% (IRR = 1.51; 95% CI: 1.20–1.90), indicating that physical activity could be an interactor but not a confounder. We observed the same pattern for perceived stress and alcohol drinking. However, the stratum-specific IRRs of smokers (IRR = 1.80; 95% CI: 1.05–2.81) and non-smokers (IRR = 1.52; 95% CI: 1.35–1.62) were different from each other, and both were larger than the crude estimate, which indicates that smoking can be both a confounder and an interactor in the association between physical well-being and chronic pain. The IRR of the association between physical well-being and chronic pain changed slightly less than 10% when it was adjusted for smoking (IRR = 1.59; 95% CI: 1.39–1.62), which favors the explanation that smoking is not a strong confounder of this association. Furthermore, the magnitude of the association between physical well-being and chronic pain in low sleep quality (IRR = 1.54, 95% CI: 1.01–2.26) is not different from that in high sleep quality (IRR = 1.52, 95% CI: 0.97–2.20). The crude estimate of this association and the stratum-specific estimates are similar, indicating that sleep quality is neither a confounder nor an interactor in the association between physical well-being and chronic pain.

### 3.4. Interaction Analysis

Interaction analysis (Table 4) showed that the RERI of physical well-being and physical activity on pain was 0.25 (95% CI: 0.13–0.60), which indicates that the joint effect on the additive scale of physical activity and physical well-being together was greater than the sum of the effects of physical activity alone and physical well-being alone. Moreover, we found that low physical well-being, when it is present together with no alcohol consumption, shows an excess risk of pain (RERI = 0.11; 95% CI: 0.06–0.36).

The interaction results did not show any indication of departure from the additivity of the effects in other covariates. AP and the Synergy index gave results that were consistent with those shown by RERI.

## 4. Discussion

Although previous research discussed the relation between biopsychosocial and lifestyle factors and chronic pain [40,41,42,43,44,45], to our knowledge, this is the first longitudinal study of chronic pain incidence among students in a high-prevalence region that examines the potential mediators, interactors, and confounders. 

The high incidence of chronic pain found in this study is compatible with that forecasted by national reports for the Galician region [8]. As mentioned before, Galicia is an extremely humid region with low barometric pressure, a characteristic that some authors linked with a high frequency of chronic pain [9]. Some animal studies showed that lower pressure might stimulate the sympathetic nervous system and activate pain fibers [46]. Moreover, humidity can affect the perceived temperature rather than the actual temperature by itself and, thus, decrease a person’s comfort, which might result in more pain perception [47]. 

It was reported that Galicia has the highest suicide rates in Spain among people under 30 [48]. Research linked this issue to unemployment during economic recession periods [49].

In this study, perceived stress played an important role in mediating the effect of physical well-being on chronic pain onset. We showed that approximately one-seventh of the total effect of physical well-being on chronic pain development was mediated by perceived stress.

The role of mediator played by perceived stress may relate to the fact that students with higher physical well-being levels have better social support and a greater sense of individual cohesion to overcome stressors and life changes [50]. Lower physical well-being is then expected to increase these individuals’ stress levels. Higher stress levels induce lower thresholds of pain perception due to impaired dopaminergic activity in the nucleus accumbens and, consequently, may cause hyperalgesia [51,52,53]. Moreover, the level of perceived stress in our study population was rather low, so it is plausible that higher levels of stress could reveal a stronger indirect effect between physical well-being and chronic pain through perceived stress.

Due to the physiological overlap that pain has with stress [54] and physical well-being [16], it would be reasonable to suspect that students who perceived more stress or lower physical well-being at baseline might have had chronic pain before starting the study; this may have caused potential reverse causation in our research. In addition, perceived stress could represent both a cause and a consequence of low physical well-being [55]. This bidirectional association between physical well-being and stress can create a vicious cycle by promoting pain perception in students. Although our design was longitudinal, the aforementioned reciprocal inter-relation could be compatible with a reverse causation procedure between mediator and exposure, since they were both collected at baseline.

Furthermore, our results suggest that physical activity interacts with physical well-being and that students with low physical well-being and low physical activity levels are at higher risk of developing chronic pain. Physical activity and exercise reduce the excessive sensitivity of central neurons by altering the neuroimmune signals in the central nervous system and increasing the release of endogenous opioids and serotonin in the brainstem pain inhibitory pathways [56,57,58].

Moreover, in this study, abstainers/low drinkers who had low physical well-being experienced more chronic pain than other subgroups. The analgesic effects of alcohol, described in previous studies, could explain this feature. Consuming a moderate amount of alcohol can increase the level of the neurotransmitters in the central nervous system that are responsible for reducing the activity of neurons and decreasing pain experience [35,45,59].

Stratum-specific analysis showed that, in our study, smoking could be both a confounder and an interactor of the association between physical well-being and chronic pain. However, both interaction and confounding analyses showed that the impact of smoking in this association is so small that it can be dismissed.

The interrelation between psychosocial factors may also explain the results of this study. For example, low physical well-being among those who smoke [60] or have low physical activity [61] deteriorates stress-coping skills by limiting adaptive coping strategies. Moreover, non-drinkers who have low physical well-being may experience more stress than moderate drinkers, as there is evidence that moderate alcohol consumption can reduce the body’s stress response [62,63]. As we showed, higher perceived stress can be responsible for part of the total effect between physical well-being and chronic pain.

### Limitations

The longitudinal character of the study supports the belief that the direct and indirect (through perceived stress) association between physical well-being and chronic pain may be causal. However, our results should be interpreted while considering some limitations. First, we should consider that pain status was assessed using a self-report questionnaire. Although the perception of pain is not clinically measurable, it is possible that using an active clinical examination for measuring the outcome could lead to other potential associations. Second, other potential confounder variables, such as personality traits and depression, which could give a broader vision of the biopsychosocial model approach, were not included. Finally, this longitudinal study measured exposure and mediators at baseline without lapses, and the temporal ambiguity between exposure (physical well-being) and mediator (perceived stress) cannot be disregarded. It is important to use caution when applying the findings of this study to a broader population, as the research was conducted on a specific sample.

Knowing the role of covariates in the relation between subjective exposures and outcomes is of paramount importance. For example, adjusting a mediator variable can block a causal path, and distinguishing a confounder from an interactor provides a more accurate interpretation of the results.

In conclusion, in a region with an a priori high risk of chronic pain, perceived stress, as an emotional state, plays a relevant role in the relation between physical well-being and pain and might be regarded as an intermediate outcome for evaluating interventions aimed at reducing chronic pain in students. If perceived stress is elected as a high-priority intervention in pain management, physical well-being should be acknowledged as one of the determinants of perceived stress. Furthermore, these findings may enable professionals to distinguish high-risk from low-risk subgroups, focusing their interventions on subjects with lower physical well-being and levels of physical activity.

## Figures and Tables

**Table 1:** Baseline characteristics of 1024 students, Pain Study Online, Spain, 2019–2020.

Exposure	Physical Well-Being (Physical Component Summary)	
	Low Mean = 48.4 (*n* = 154)	High Mean = 58.07 (*n* = 460)
Age; mean (SD)	24.95 (6.2)	24.61 (6.1)
Sex; *n* (%)		
Male	37 (24.50)	93 (20.53)
Female	114 (75.50)	360 (79.47)
Physical activity; *n* (%)		
High	34 (21.12)	127 (78.88)
Low	108 (25.29)	319 (74.71)
Perceived stress; *n* (%)		
High (>8)	88 (26.91)	239 (73.09)
Low (<8)	41 (17.01)	200 (82.99)
Smoking; *n* (%)		
Non-current smokers	112 (22.72)	381 (77.28)
Current smokers	42 (34.71)	79 (65.29)
Alcohol consumption; *n* (%)		
Abstainers/low drinkers	25 (25.77)	72 (74.23)
Moderate drinkers	30 (21.90)	107 (78.10)
Sleep quality; *n* (%)		
Low >5	56 (52.83)	149 (36.97)
High <5	50 (47.17)	254 (63.03)

**Table 2:** Natural direct, indirect, and total effects of physical well-being on chronic pain incidence among students, Pain Study Online, Spain, 2019–2020.

Mediator	Unadjusted				Adjusted ^a^			
	Natural Indirect Effect	Natural Direct Effect	Natural Total Effect	Proportion Mediated, %	Natural Indirect Effect	Natural Direct Effect	Natural Total Effect	Proportion Mediated, %
	IRR (95% CI)	IRR (95% CI)	IRR (95% CI)		IRR ^a^ (95% CI)	IRR ^a^ (95% CI)	IRR ^a^ (95% CI)	
**Physical activity *(low vs. high)***	0.97 (0.87, 1.07)	0.64 (0.45, 0.79)	0.60 (0.44, 0.81)	5.1	0.96 (0.85, 1.09)	0.63 (0.50, 0.82)	0.59 (0.49, 0.82)	6.4
**Perceived stress *(low vs. high)***	0.94 (0.91, 1.03)	0.63 (0.45, 0.77)	0.59 (0.46, 0.92)	9.3	0.92 (0.89, 1.00)	0.64 (0.48, 0.81)	0.58 (0.50, 0.81)	12.5
**Smoking *(non-current vs. current)***	0.98 (0.89, 1.05)	0.62 (0.45, 0.80)	0.60 (0.44, 0.80)	3.2	0.97 (0.84, 1.07)	0.63 (0.49, 0.79)	0.60 (0.50, 0.78)	4.8
**Drinking** ***(abstainers/low vs. moderate)***	1.02 (0.86, 1.15)	0.63 (0.44, 0.80)	0.60 (0.48, 0.82)	−3.5	1.03 (0.83, 1.16)	0.63 (0.48, 0.78)	0.65 (0.50, 0.79)	−5.3
**Sleep quality *(good vs. poor)***	0.98 (0.84, 1.10)	0.63 (0.48, 0.81)	0.61 (0.47, 0.80)	3.3	0.98 0.93, 1.07)	0.64 (0.48, 0.84)	0.62 (0.49, 0.85)	3.4

Abbreviations: CI, confidence interval; IRR, incidence rate ratio. ^a^ Adjusted for age and sex.

**Table 3:** Stratum-specific incidence rate ratios of the association between physical well-being and chronic pain among students, Pain Study Online, Spain, 2019–2020.

Covariates	Stratum-Specific IRR	IRR Crude	IRR Pooled
Low physical activity	1.76 (1.28, 2.36)	1.49 (1.24, 1.80)	1.51 (1.20, 1.90)
High physical activity	1.20 (1.06, 1.45)		
Low stress	1.27 (1.30, 1.79)	1.49 (1.24, 1.80)	1.47 (1.16, 1.86)
High stress	1.68 (1.22, 2.32)		
Non-current smokers	1.80 (1.05, 2.81)	1.49 (1.24, 1.80)	1.59 (1.39, 1.62)
Current smokers	1.52 (1.35, 1.62)		
Low/non-drinkers	1.59 (1.19, 2.75)	1.49 (1.24, 1.80)	1.44 (1.22, 1.58)
Moderate drinkers	1.44 (1.10, 1.58)		
Low sleep quality	1.54 (1.01, 2.26)	1.49 (1.24, 1.80)	1.50 (1.13, 1.98)
High sleep quality	1.52 (0.97, 2.20)		

Abbreviations: CI, confidence interval; IRR, incidence rate ratio.

**Table 4:** Measures of additive interaction between covariates and physical well-being among students in the occurrence of chronic pain, Pain Study Online, Spain, 2019–2020.

Interaction	Adjusted * IRR (95% CI)	Univariate RERI (95% CI)	Adjusted RERI * (95% CI)	AP (95% CI)	S (95% CI)
**Physical activity/physical well-being**		0.25 (0.13, 0.60)	0.27 (0.12, 0.51)	0.19 (0.13, 0.58)	3.79 (1.29, 7.84)
High activity, high well-being	1 (ref)				
Low activity, high well-being	0.89 (0.77, 1.04)				
High activity, low well-being	1.20 (1.06, 1.45)				
Low activity, low well-being	1.34 (1.07, 1.59)				
**Perceived stress/physical well-being**		0.02 (−0.89, 0.16)	0.01(−0.92, 0.17)	0.02(−0.83, 0.23)	1.07 (0.59, 1.27)
Low stress, high well-being	1 (ref)				
High stress, high well-being	1.03 (0.73, 1.09)				
Low stress, low well-being	1.27 (1.30, 1.79)				
High stress, low well-being	1.37 (0.79, 1.10)				
**Smoking status/physical well-being**		−0.07 (−0.15, 0.01)	−0.04 (−0.24, 0.10)	−0.08 (−0.10, 0.11)	0.64 (0.32, 0.70)
Non-current smokers, high well-being	1 (ref)				
Smokers, high well-being	0.74 (0.45, 1.30)				
Non-current smokers, low well-being	1.52 (1.35, 1.62)				
Smokers, low well-being	1.17 (0.92, 1.26)				
**Drinking status/physical well-being**		0.11 (0.06, 0.36)	0.09 (0.04, 0.28)	0.08 (0.05, 0.30)	1.81 (1.51, 2.12)
Moderate drinkers, high well-being	1 (ref)				
Abstainers, high well-being	0.68 (0.56, 0.81)				
Moderate drinkers, low well-being	1.44 (1.10, 1.58)				
Abstainers, low well-being	1.24 (1.12, 1.36)				

Abbreviations: CI, confidence interval; IRR, incidence rate ratio. * Adjusted for age and sex.

## Data Availability

Dataset and codes are available at https://osf.io/djt8f/ under the heading “Physical well-being and chronic pain”; DOI 10.17605/OSF.IO/DJT8F. [accessed on 21 March 2023].
